# Highly Sensitive Acetone Gas Sensors Based on Erbium-Doped Bismuth Ferrite Nanoparticles

**DOI:** 10.3390/nano12203679

**Published:** 2022-10-20

**Authors:** Xiaolian Liu, Jing Li, Lanlan Guo, Guodong Wang

**Affiliations:** School of Physics and Electronic Information Engineering, Henan Polytechnic University, Jiaozuo 454003, China

**Keywords:** bismuth ferrite nanoparticles, Er doping, acetone gas sensor, morphotropic phase boundary

## Abstract

The acetone-sensing performance of BiFeO_3_ is related to structural phase transformation, morphology and band gap energy which can be modulated by rare-earth ions doping. In this work, Bi_1−x_Er_x_FeO_3_ nanoparticles with different amounts of Er doping were synthesized via the sol-gel method. The mechanism of Er doping on acetone-sensing performance of Bi_1−x_Er_x_FeO_3_ (x = 0, 0.05, 0.1 and 0.2) sensors was the focus of the research. The optimal working temperature of Bi_0.9_Er_0.1_FeO_3_ (300 °C) was decreased by 60 °C compared to BiFeO_3_ (360 °C). The Bi_0.9_Er_0.1_FeO_3_ sample demonstrated the optimal response to 100 ppm acetone (43.2), which was 4.8 times that of pure BFO at 300 °C. The primary reason, which enhances the acetone-sensing performance, could be the phase transformation induced by Er doping. The lattice distortions induced by phase transformation are favorable to increasing the carrier concentration and mobility, which will bring more changes to the hole-accumulation layer. Thus, the acetone-sensing performance of Bi_0.9_Er_0.1_FeO_3_ was improved.

## 1. Introduction

Acetone is a common volatile organic compound that is widely used in the chemical industry and laboratories. However, long-term exposure to acetone or excessive inhalation of acetone causes great damage to the skin, nervous system, and breathing system, which leads to dermatitis, headache, nausea and coma [1]. Thus, the detection of acetone is highly significant for human health. Meanwhile, the detection of acetone can be used in the diagnosis of some diseases. For example, in the breath of diabetes patients, the concentration of acetone is more than twice as much as that of healthy people [2]. Compared to blood tests, acetone gas sensing is quick, easy, and noninvasive. Consequently, great attention has been paid to the study of acetone gas sensors. Plenty of oxides have been reported to show acetone-sensing performance, such as ZnO, SnO_2_, Co_3_O_4_, Fe_2_O_3_, WO_3_ and so on. Some strategies have been adopted to optimize gas sensing performance, such as designing nanomaterial with special morphology and structure [3,4], preparation of composite materials [5], doping with rare-earth and noble-metal ions [6,7] and decorating with other nanomaterial [8]. However, studies have shown that binary metal oxides display excellent gas sensing performance, but suffer from high operating temperature and relatively poor stability [9,10]. Consequently, the study of the acetone gas sensor based on ternary oxides has been of great interest to researchers due to the good thermal stability and easy modulation of sensing performance.

BiFeO_3_ (BFO) is a typical ABO_3_ perovskite material with a rhombohedral structure (*R*3c space group). Studies have been carried out for its applications in magnetoelectric memory, photovoltaic devices, spintronics devices and photocatalysis [11,12,13,14]. Researchers also reported the gas sensing performance of BFO to acetone, formaldehyde, oxygen, nitrogen dioxide, isopropanol and so on [15,16,17,18,19]. Xu et al. [20] have studied the gas sensing of BFO nanocrystals to isopropanol. At 240 °C, the response to 100 ppm isopropanol is 31 and the response and recovery time are 6 s and 17 s. Yu et al. [21] have prepared a hierarchical BFO microcubes-based sensor. The response to 200 ppm acetone is about 5.2 at 240 °C, and the response and recovery times are 10 s and 9 s. Chakraborty et al. [22] have prepared BFO nanoparticles and studied the acetone sensing properties. The response of the prepared sensor to 1 ppm acetone is about 1.8 with great long-term stability of 365 days. According to previous studies, the mechanism for enhanced gas-sensing performance is still controversial. Neogi et al. [23] have prepared an acetone sensor based on yttrium-doped BFO powder, and the prepared sensor shows significant enhancement in response to 200 ppm acetone (~52) and great selectivity and stability for at least 300 days. The primary reason for the enhancement of sensing behavior is the structure and morphology changes induced by moderate yttrium doping. Bala et al. [19] have fabricated Ca^2+^-doped BFO thin films, and more oxygen vacancy is obtained through doping which enhanced the H_2_ sensing characteristics. Sobhan et al. [24] have studied the O_2_ sensing performance of Ni^2+^-doped and Pb^2+^-doped BFO nanofibers, and claimed that the minor carrier compensation is the key factor affecting gas-sensing performance. Peng et al. [25] have reported a BFO acetone sensor with the ppb detection limit and concluded that the improvement in gas sensitivity is attributed to the morphotropic phase boundary induced by La^3+^ doping. Meanwhile, factors which can improve the gas sensitivity of the BFO sensor are numerous. Tong et al. [17] have reported the gas-sensing performance of BFO nanoparticles had strong dependency on the particle size and morphology. Douani et al. [26] reported that adding carbon fibers can elevate the humidity-sensing property of BFO nanoparticles. Therefore, research should be undertaken on the performance-optimization and enhancement mechanism of BFO gas sensors.

According to earlier reports, the acetone-sensing performance of BFO is related to the structural phase transformation, morphology, and band gap energy which can be modulated by rare-earth ions doping. Herein, Bi_1−x_Er_x_FeO_3_ particles were synthesized via the sol-gel process and then Bi_1−x_Er_x_FeO_3_ sensors were fabricated and studied.

## 2. Experiments

### 2.1. Synthesis of Bi_1−x_Er_x_FeO_3_ Nanoparticles

The sol-gel method was used for nanoparticles synthesis. First, Bi(NO_3_)_3_⋅5H_2_O and Er(NO_3_)_3_⋅6H_2_O were dissolved in 10 mL acetic acid at the desired stoichiometry, and stirred for half an hour in a water bath heated to 45 °C. Fe(NO_3_)_3_⋅9H_2_O was dissolved in 10 mL 2-methoxytehanol with the same approach. Then, the two solutions were blended and stirred uniformly via the above method to acquire 0.3 M sol. Finally, the prepared sol was annealed at 625 °C for 30 min to obtain Bi_1−x_Er_x_FeO_3_ nanoparticles.

### 2.2. Materials Characterization

The structure of Bi_1−x_Er_x_FeO_3_ was analyzed by X-ray diffraction (XRD, Smart Lab, Rigaku, Tokyo, Japan). The morphology of Bi_1−x_Er_x_FeO_3_ nanoparticles were observed by a field-emission scanning electron microscope (SEM, Zeiss Gemini 300, Oberkochen, Germany). The UV-Vis absorption spectrum was measured using a UV-vis spectrophotometer (UV-3600 plus, Shimadzu, Kyoto, Japan). The composition and element valence were characterized by X-ray photoelectron spectroscopy (XPS, Thermo Scientific K-Alpha, Waltham, MA, USA).

### 2.3. Measurement of Bi_1−x_Er_x_FeO_3_ Gas Sensors

Bi_1−x_Er_x_FeO_3_ gas sensors were fabricated by mixing Bi_1−x_Er_x_FeO_3_ nanoparticles with moderate ethanol to form a smooth slurry, and then spreading the slurry on a ceramic plate on which Ag-Pd interdigital electrodes had been made. Then, the sensors were placed into a muffle furnace (300 °C) for 2 h. Gas-sensing performance was characterized via a multifunctional gas-sensing test system (CGS-MT, Beijing Elite Tech Co., Ltd., Beijing, China). The definition of the sensing response can be expressed by *S* = *R*_g_/*R*_a_, and *R*_g_ is the resistance of the sensor in gas. Accordingly, *R*_a_ is the resistance in air.

## 3. Results

### 3.1. Structure

**Figure 1:**
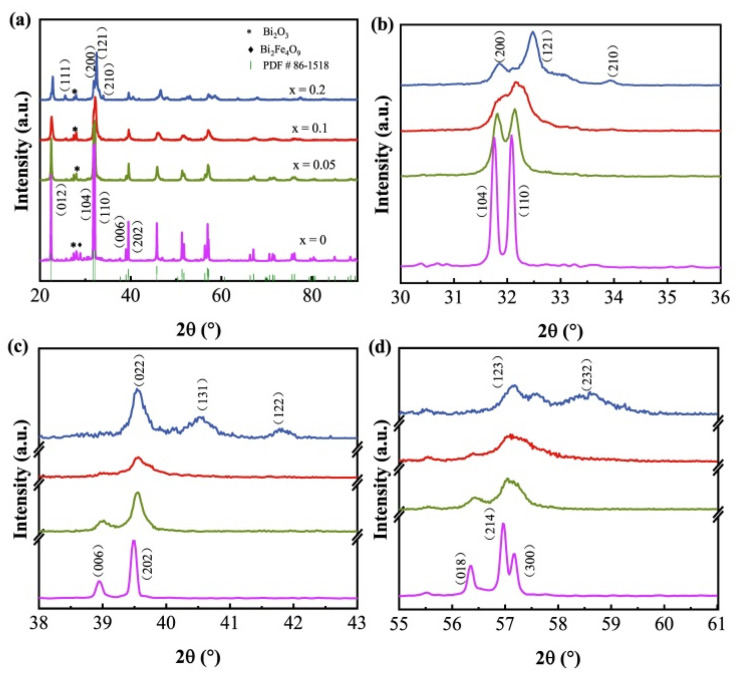
(**a**) XRD patterns of Bi_1−x_Er_x_FeO_3_ nanoparticles, and the enlarged diffraction peaks (**b**) around 32°, (**c**) ranging from 38° to 43°, (**d**) ranging from 55° to 61°.

XRD patterns of Bi_1−x_Er_x_FeO_3_ nanoparticles were shown in the Figure 1a. When x = 0, BiFeO_3_ nanoparticles have a rhombohedral perovskite structure (JCPDS No. 86-1518). The (104) and (110) diffraction peaks are broadened as the Er-doping content increases in Figure 1b. According to Debye-Scherrer’s formula, the grain size decreases as the full width at half maximum increases. Therefore, it can be speculated that Er doping has led to the reduction of the grain size of nanoparticles, which is also confirmed in the SEM figures. Since the smaller Er^3+^ ions (1.004 Å, CN8) have substituted the larger Bi^3+^ ions (1.17 Å, CN8) which decreases cell parameters and volume, the diffraction peaks of (104) and (110) shift to a higher angle. Meanwhile, they tend to merge to one peak for x = 0.1. In addition, an extra diffraction peak around 25.6° appears in the x = 0.2 sample according to Figure 1a, which indicates phase transformation has occurred. Figure 1c is the enlarged diffraction peak around 39°. The BFO nanoparticles show two diffraction peaks at 38.9° and 39.5°, corresponding to (006) and (202) diffraction peaks of rhombohedral phase, respectively. With the increase of Er-doping content, (006) and (202) gradually merged into one peak for x = 0.2. New diffraction peaks appear around 40° and 42° for x = 0.2. Figure 1d shows the enlarged diffraction peak in the range of 55–61°. The (018) diffraction peak gradually disappeared, and the (214) and (300) merge, and a new diffraction peak was observed at 59° for x = 0.2. These changes of diffraction peaks indicate that the crystal structure had transformed from *R*3*c* to *Pnma*, which is similar to earlier results [27,28]. Therefore, the diffraction peaks for x = 0.2 sample in Figure 1a are indexed to the orthorhombic phase, which is an isomorph of LaFeO_3_ (JCPDS No. 04-007-9521). In addition, minor amounts of Bi_2_Fe_4_O_9_ and Bi_2_O_3_ were observed in Bi_1−x_Er_x_FeO_3_ nanoparticles, and could be ascribed to the heterogeneity of sol during sintering [29,30].

### 3.2. Morphology

**Figure 2:**
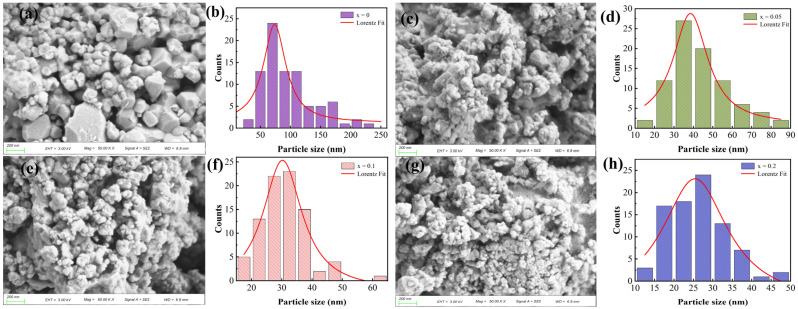
SEM images of Bi_1−x_Er_x_FeO_3_: (**a**) x = 0, (**c**) x = 0.05, (**e**) x = 0.1 and (**g**) x = 0.2. The size distribution of Bi_1−x_Er_x_FeO_3_: (**b**) x = 0, (**d**) x = 0.05, (**f**) x = 0.1 and (**h**) x = 0.2.

Figure 2 shows the SEM images of Bi_1−x_Er_x_FeO_3_ nanoparticles. The BiFeO_3_ nanoparticles show irregular shape and uneven grain size. With the increase of Er doping, nanoparticles change to spheres and the size of the nanoparticles decreases by degrees. The average grain size of nanoparticles was 128 nm, 58 nm, 37 nm, and 29 nm, respectively. The decrease of grain size is because smaller Er^3+^ ions have replaced larger Bi^3+^ ions thus increasing the nucleation rate. The decrease of particle size is conducive to a larger specific area, which can improve the gas sensitivity.

### 3.3. Band Gap Energy

**Figure 3:**
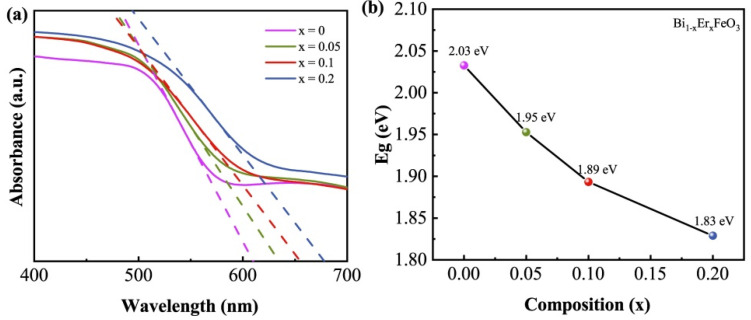
(**a**) Absorption spectra of Bi_1−x_Er_x_FeO_3_ nanoparticles. (**b**) Band gap energy (*E*_g_) as a function of Er-doping content.

UV-vis absorption spectroscopy was performed to obtain the band gap energy *E*_g_ of Bi_1−x_Er_x_FeO_3_ nanoparticles as illustrated in Figure 3a. The relationship between band gap energy *E*_g_ and absorption threshold *λ*_g_ of a semiconductor is *E*_g_ (eV) = 1240/*λ*_g_ (nm). The calculated band gap energy is shown in Figure 3b. The *E*_g_ decreases with the increase of Er content, and *E*_g_ is 2.03, 1.95, 1.89 and 1.83 eV, respectively. Earlier reports have found that smaller *E*_g_ can generate larger gas-sensing response [31,32].

### 3.4. XPS

**Figure 4:**
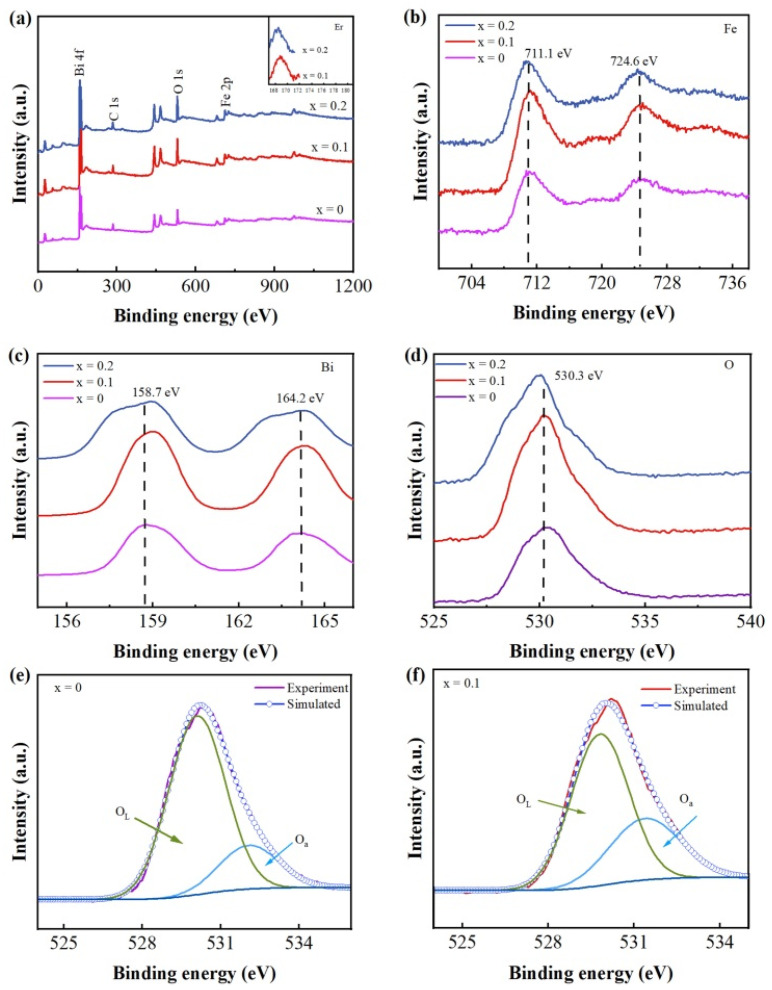
XPS spectra of (**a**) Bi_1−x_Er_x_FeO_3_ nanoparticles, (**b**) Fe 2p, (**c**) Bi 4f and (**d**) O 1s. The fitted curves of O 1s for (**e**) x = 0, (**f**) x = 0.1.

Figure 4a is the XPS spectra of Bi_1−x_Er_x_FeO_3_ nanoparticles. The Bi, Fe, O and C were observed. The inset of Figure 4a shows the peak around 168 eV which is attributed to the Er 4d level of Er^3+^ ions. In Figure 4b, peaks at 711.1 eV and 724.6 eV correspond to the Fe 2p_3/2_ level and Fe 2p_1/2_ level of the Fe^3+^ state. The two peaks at 158.7 eV and 164.2 eV in Figure 4c are attributed to the Bi 4f_7/2_ and Bi 4f_5/2_ levels of Bi^3+^ ions. Figure 4d is the XPS spectrum of O 1s. The O 1s spectra of the x = 0 and x = 0.1 samples have been fitted as shown in Figure 4e and Figure 4f. The 530.1 eV peak and 532.1 eV are assigned to the lattice oxygen (denoted by O_L_) and absorbed oxygen (denoted by O_a_), respectively. The percentage of O_a_ in x = 0.1 is higher than in x = 0 which suggests Er doping can increase the absorption of oxygen and is beneficial for gas-sensing behavior.

### 3.5. Gas-Sensing Performance

**Figure 5:**
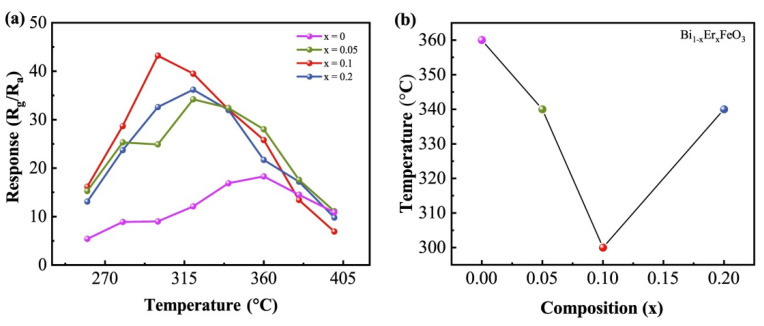
(**a**) Temperature dependence of response of Bi_1−x_Er_x_FeO_3_ to 100 ppm acetone. (**b**) The optimal operating temperature as a function of Er content.

Figure 5a is the relation between response to acetone and temperatures. It can be found that Bi_0.9_Er_0.1_FeO_3_ shows the largest response at 300 °C and the responses first rise and then fall. This is because the response of the sensor to acetone gas depends on the relative balance between the adsorption and desorption of the acetone gas and the reaction with the adsorbed oxygen [33,34]. Reaction between acetone molecules and adsorbed oxygen lacks sufficient energy under low temperature, which leads to a poor response. However, the energy of acetone molecules increases because of the increase in temperature, which provides enough energy needed for the reaction, so the response is enhanced. When the temperature increases further, desorption of acetone molecules increases and the acetone absorbed on the nanoparticles is reduced, thus the response is decreased. The optimal operating temperature of Er-doped sensors is lower than that of undoped one (360 °C), as shown in Figure 5b, which demonstrates that Er doping can decrease the optimal operating temperature effectively. The following test was carried out at 300 °C.

**Figure 6:**
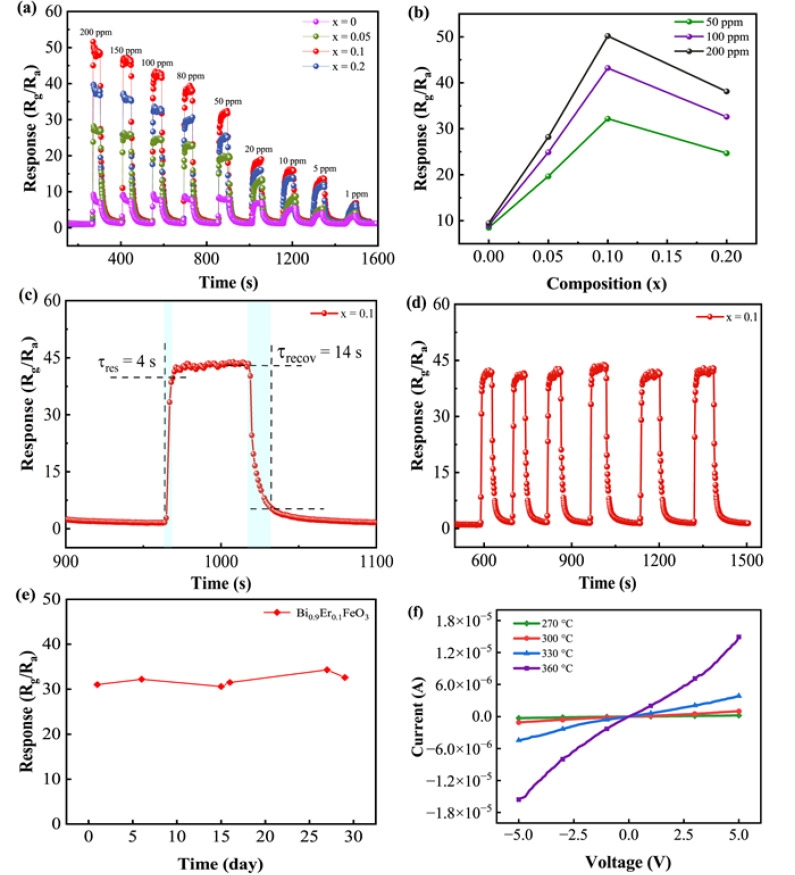
(**a**) Dynamic response and recovery curves of Bi_1−x_Er_x_FeO_3_ sensors to acetone. (**b**) Response of sensors as a function of Er-doping content. (**c**) Transient response of Bi_0.9_Er_0.1_FeO_3_ sensor to 100 ppm acetone. (**d**) The response to 100 ppm acetone measured for six cycles. (**e**) Stability measurement of Bi_0.9_Er_0.1_FeO_3_ sensor to 50 ppm acetone within a month. (**f**) Current-voltage characteristics of Bi_0.9_Er_0.1_FeO_3_ sensor at different temperatures.

Figure 6a shows the response of sensors to acetone for different concentration (1, 5, 10, 20, 50, 80, 100, 150 and 200 ppm). The prepared sensors show a low detection limit of 1 ppm to acetone. The relation curve between response and Er-doping content is shown in Figure 6b. With an increase of Er content, the response first increases then decreases, reaching a maximum for x = 0.1 sample. The responses of the Bi_0.9_Er_0.1_FeO_3_ sensor to 1 and 100 ppm acetone are 7 and 43.2 while those of the undoped BFO sensor are 3.2 and 9. The enhanced response is related to the phase transformation of the BFO induced by Er doping. Xing et al. [27] have reported that Er doping would lead to the phase transformation of Bi_1−x_Er_x_FeO_3_ from *R*3*c* to *Pnma* phase, and the proportion of *Pnma* phase increased with the increase of Er content. When x = 0.05, the proportions of *R*3*c* and *Pnma* phase are 77.6% and 22.3%, respectively. When x = 0.1, the proportions are 58.1% and 41.9%, respectively, and the ratio of the two phase is about 1:1. That means the x = 0.1 sample is at the so-called morphotropic phase boundary (MPB) and has the largest lattice distortion. When x = 0.2, only *Pnma* phase exists. According to a previous study, large lattice distortion will decrease the electron effective mass and scattering probability. The carrier concentration and electron mobility are inversely proportional to the two parameters. The increase of carrier concentration and electron mobility will lead to a thicker hole-accumulation layer at the surface of the nanoparticles, and bring more changes for the accumulation layer. Therefore, the changes in resistance of the senor increase and the acetone-sensing performance is improved. This phenomenon has also been reported in the La^3+^-doped BFO gas sensor [25].

Meanwhile, the morphology of nanoparticles is considered to have an effect on the response of the Bi_1−x_Er_x_FeO_3_ sensor. The specific area of nanoparticles increases and more active sites generate, thus more gas molecules will be absorbed. That explains why all doped samples show larger responses than the pure BFO. However, the x = 0.2 sample shows the smallest grain size while the response is not the largest. As a result, we conclude that morphology is not the dominating factor to improve acetone-sensing performance for Bi_1−x_Er_x_FeO_3_ sensors. In addition, Er doping has decreased the band gap energy of nanoparticles which may have contributed to the enhancement of the acetone-sensing response.

The transient response of the Bi_0.9_Er_0.1_FeO_3_ sensor to 100 ppm acetone is shown in Figure 6c. Response time (*τ*_res_) refers to the amount of time that the resistance of the Bi_1−x_Er_x_FeO_3_ sensor goes from *R*_a_ to *R_a_* + | *R_a_*-*R_g_* | × 90%. Recovery time (*τ*_recov_) is defined as the time needed from *R*_g_ to *R_g_* − | *R_a_*-*R_g_* | × 90%. The *τ*_res_ and *τ*_recov_ of the Bi_0.9_Er_0.1_FeO_3_ sensor are about 4 s and 14 s, which is close to an earlier report [17]. Figure 6d is the response of the Bi_0.9_Er_0.1_FeO_3_ sensor to 100 ppm acetone measured for six cycles, demonstrating good stability. The stability of the Bi_0.9_Er_0.1_FeO_3_ sensor to 50 ppm of acetone was measured randomly within a month (on day 1, 6, 15, 16, 27 and 29) as shown in Figure 6e. The measurement was carried out at 300 °C. The response has changed little in a month, suggesting the great long-term stability of the prepared gas sensor. Figure 6f is the current-voltage (*I*-*V*) characteristics of the Bi_0.9_Er_0.1_FeO_3_ sensor tested at 270–360 °C. The current is linearly varying with voltage. Thus the conduction mechanism of the sensor is an ohmic conduction mechanism and the key factor in gas sensing is Bi_0.9_Er_0.1_FeO_3_ nanoparticles. Meanwhile, the *I*-*V* curves show no deformation as the operating temperature increases which means the prepared sensor can be applied at high temperatures.

**Figure 7:**
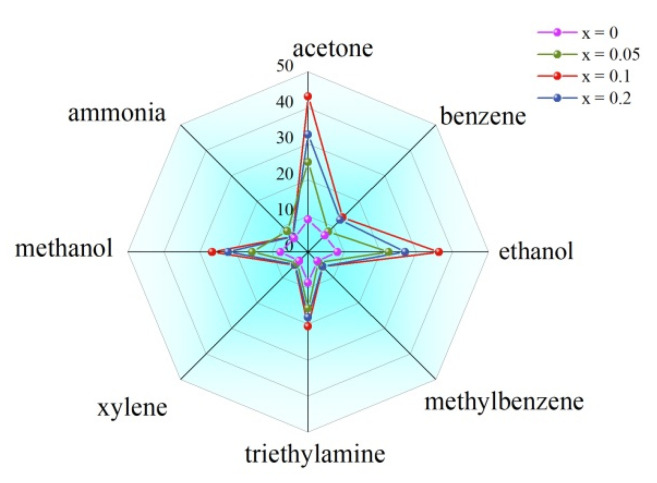
Selectivity of Bi_1−x_Er_x_FeO_3_ sensors to different gases.

The selectivity of gas sensors has been characterized by measuring responses in acetone, methanol, ethanol, benzene, methylbenzene, xylene, ammonia and triethylamine (concentration = 100 ppm) as seen in Figure 7. The undoped BFO sensor demonstrated the minimum differences of responses to all gases thus the worst selectivity. The x = 0.1 sample shows the largest response to all gases, and the response to acetone was significantly higher than the others. For all sensors, the response to acetone is the largest followed by alcohols, triethylamine and ammonia, and the response to benzene and its derivatives are minimum.

## 4. Discussion

**Figure 8:**
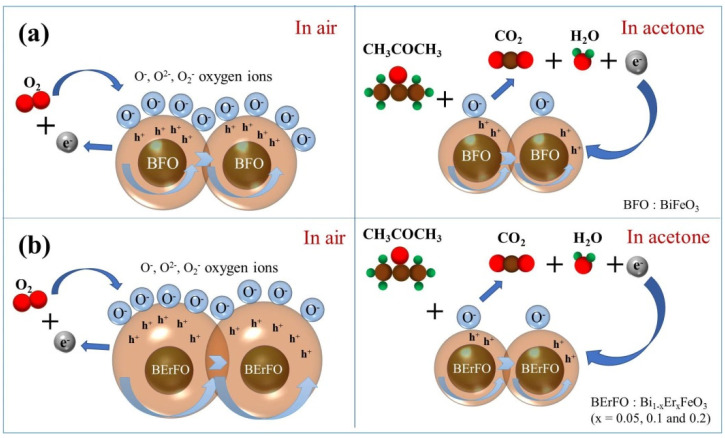
The illustration of the gas-sensing mechanism of Bi_1−x_Er_x_FeO_3_ sensors: (**a**) x = 0, (**b**) x = 0.05, 0.1, 0.2.

Figure 8 illustrates the possible mechanism and the principle is explained by Equations (1)–(5), where O^−^, e^−^ and h^+^ represent oxygen ions, electrons and holes, respectively. BFO and Er-doped BFO are generally considered to be p-type semiconductors. As shown in Figure 8a, in air, O_2_ is absorbed on the BFO nanoparticles and captures electrons from particles, forming O^−^. Thus, a hole-accumulation layer is obtained at the surface (Equations (1) and (2)). Consequently, the energy band is lowered at the surface, resulting in a decrease in resistance. When BFO sensors are exposed in reducing air acetone, the acetone molecule will react with O^−^ to release electrons to nanoparticles. Those released electrons recombine with holes and the resistance increases (Equations (3)–(5)).

O_2(gas)_ = O_2(ads),_ (1)

O_2(ads)_ + 2e^−^ = 2O^−^_(ads),_ (2)

CH_3_COCH_3(gas)_→CH_3_COCH_3(ads),_ (3)

CH_3_COCH_3(ads)_ + 8O^−^_(ads)_→3CO_2_ + 3H_2_O + 8e^−^, (4)

h^+^ + e^−^ = null. (5)

When BFO nanoparticles are doped with Er^3+^ ions, phase transformation and lattice distortion occur which increase the carrier concentration and mobility. Consequently, recombination of electrons and holes is reduced and the hole-accumulation layer is widened. As a result, more oxygen will be absorbed. When the Bi_1−x_Er_x_FeO_3_ sensor is exposed to acetone, larger changes of resistance and responses are obtained, as shown in Figure 8b.

## 5. Conclusions

Bi_1−x_Er_x_FeO_3_ nanoparticles were synthesized via the facile sol-gel process. Er doping has resulted in the transformation from rhombohedral phase to orthorhombic phase. Meanwhile, the nanoparticle size and band gap energy decreased with the increase of Er-doping content, which is beneficial for the improvement of acetone-sensing performance. The Bi_0.9_Er_0.1_FeO_3_ is located at the MPB and displays the optimum response to 100 ppm acetone (43.2) at 300 °C, which is 4.8 times that of pure BFO. In addition, the Bi_0.9_Er_0.1_FeO_3_ sensor shows fast response and recovery, the *τ*_res_ and *τ*_recov_ of the Bi_0.9_Er_0.1_FeO_3_ sensor are about 4 s and 14 s. The phase transformation is considered to have played the key role in the enhanced acetone-sensing performance for the Bi_1−x_Er_x_FeO_3_ sensors.

## Data Availability

Not applicable.
